# Severe Hypomagnesemia Caused by Proton-Pump Inhibitors in a Patient With an Ostomy

**DOI:** 10.7759/cureus.55856

**Published:** 2024-03-09

**Authors:** Kabeer Ali, Aleem A Ali, Vishal Jaikaransingh

**Affiliations:** 1 Internal Medicine, University of Florida College of Medicine – Jacksonville, Jacksonville, USA; 2 Gastroenterology and Hepatology, University of Florida College of Medicine – Jacksonville, Jacksonville, USA; 3 Nephrology, University of Florida College of Medicine – Jacksonville, Jacksonville, USA

**Keywords:** trpm6 gene, trpm6, ilesotomy, ostomy, proton-pump inhibitor, ppi, refractory hypomagnesemia

## Abstract

Proton pump inhibitors (PPIs) are commonly used for many gastrointestinal issues, such as gastroesophageal reflux disease (GERD), peptic ulcer disease, and Zollinger-Ellison syndrome. Many patients are on life-long daily therapy with this class of medications. The adverse effects of long-term use of PPI have been studied, and over the last two decades, a link between hypomagnesemia and PPI has been established. In addition, other electrolyte derangements can also ensue, such as hypokalemia and hypocalcemia. Losses through the gastrointestinal or renal systems may also be responsible for this electrolyte disturbance. In this case, we present a "perfect storm" of a patient who, in addition to having ongoing gastrointestinal losses through an ostomy, had severe hypomagnesemia to less than 1 mg/dL compounded by PPI use. Through its unique mechanism of action on intestinal epithelial cells, PPI use in certain settings can potentially be catastrophic. Severe hypomagnesemia may manifest as tetany, convulsions, tremors, arrhythmias, or torsades de pointes.

## Introduction

Proton pump inhibitors (PPIs) are indicated for gastroesophageal reflux disease (GERD), peptic ulcer disease, and for prophylaxis against gastric ulcers in patients on prolonged courses of corticosteroids and nonsteroidal anti-inflammatory drugs. The association between PPI therapy and hypomagnesemia has been recognized since 2006 [1]. PPIs are believed to decrease the activity of transient receptor potential melastatin (TRPM6), which causes reduced magnesium absorption [2]. In most patients, the decrease in serum magnesium is found incidentally on routine lab testing but does not affect the patient clinically. However, in those with additional causes of magnesium loss, such as loop diuretics, thiazide diuretics, and significant gastrointestinal losses, the effect on serum magnesium levels is exacerbated. Severe hypomagnesemia can be catastrophic and lead to arrhythmias, seizures, and sudden cardiac death. This case describes PPI use causing severe, persistent hypomagnesemia. Hypomagnesemia was intermittently improved on oral replacement therapy, but the patient had recurrent presentations to the hospital with severe hypomagnesemia requiring intravenous magnesium therapy. On withdrawal of PPI therapy, the patient’s magnesium normalized. This case was previously presented as a meeting abstract at the 2022 Southern Medical Research Conference on February 10, 2022.

## Case presentation

A 76-year-old male with a medical history of coronary artery disease with past coronary artery bypass grafting, hepatitis C, GERD, and total colectomy with the creation of an ileostomy secondary to colitis was referred to outpatient nephrology when a routine complete metabolic laboratory panel revealed severe hypomagnesemia of 0.6 mg/dL and the patient was started on daily magnesium oxide without resolution. The patient was asymptomatic and denied any anorexia, nausea, vomiting, lethargy, weakness, personality change, tetany, tremors, or muscle fasciculations. His ostomy output was between 500 mL and 1 L per day. He was not on any diuretic therapy and denied alcohol use or any recent dietary changes.

**Figure 1:**
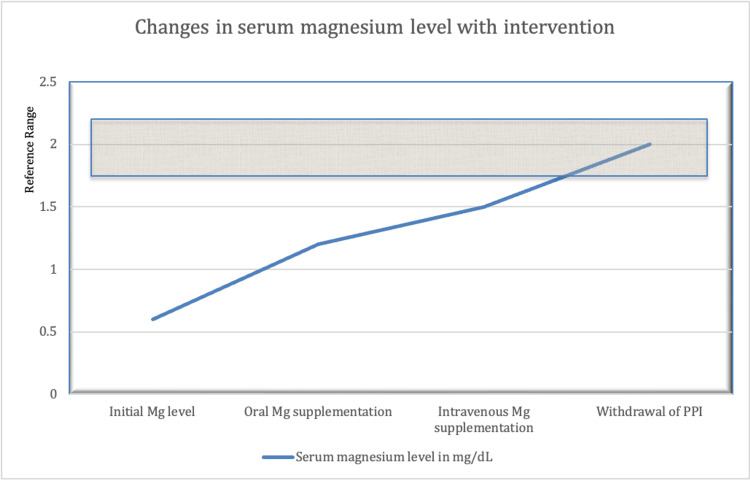
Highlighting trends in the patient's serum magnesium level This figure is the authors' own creation

On initial nephrology consultation, he was found to have persistent hypomagnesemia (range 1.2-1.3 mg/dL) despite adherence to prescribed oral magnesium oxide supplementation (Figure 1). The only recent change to his medication regimen was being started on pantoprazole for GERD. Repeat laboratory investigations ordered at that visit showed worsening severe hypomagnesemia of 0.5 mg/dL, prompting referral to the emergency department for intravenous electrolyte replacement therapy. The electrocardiogram revealed a normal sinus rhythm with no prolonged PR interval or acute ST-segment changes.

**Figure 2:**
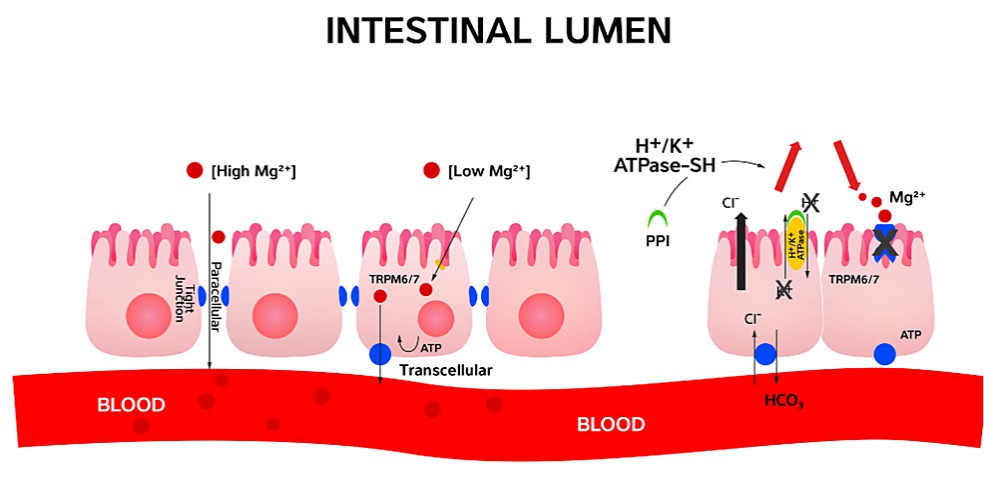
The passive paracellular transport and active transcellular transport of magnesium This figure is the authors' own creation

The patient's fractional excretion of magnesium (FeMg) was 1%, consistent with non-renal losses. The proposed route of magnesium loss was thought to be gastrointestinal, especially in an ostomy setting and PPI initiation (Figure 2). The patient was given intravenous magnesium sulfate to normalize his serum magnesium, and his PPI was discontinued and replaced with an H_2_ receptor antagonist. Subsequent chemistries revealed resolved hypomagnesemia, and the patient required no further magnesium supplementation.

## Discussion

Proton pump inhibitor therapy has been shown to result in electrolyte abnormalities, including hypomagnesemia [3]. PPI-induced hypomagnesemia was first recognized in 2006, with a report of two patients developing severe magnesium deficiency in addition to hypocalcemia and hypokalemia while on long-term PPI treatment [4].

Hypomagnesemia is related to the patient's duration of PPI therapy [5]. This patient recovered magnesium levels in just a few days after PPI discontinuation. Per a systematic review by Hess et al., the average time to recover after PPI discontinuation was only four days. Interestingly, the onset time of hypomagnesemia is very variable and can range from 14 days to 13 years. Our patient developed hypomagnesemia within a few months. Magnesium is the second-most abundant intracellular cation, and its homeostasis is intricately regulated by intestinal absorption and renal excretion [6]. Most adults who consume a Western diet are believed to be deficient in magnesium at baseline due to loss of magnesium from food processing and poor oral magnesium absorption [7]. Intestinal magnesium absorption occurs through passive paracellular transport via claudins and active transcellular transport via TRPM6. It is hypothesized that proton pump inhibitors impair active transcellular magnesium transport by inhibiting TRPM6. One retrospective clinical evaluation also looked at genetic mutations that led to a complete loss of function of TRMP6, which can cause profound primary hypomagnesemia with secondary hypocalcemia [8]. This condition usually manifests itself in early infancy. 

Severe hypomagnesemia can lead to an array of cardiac and neurologic dysfunctions, of which the most concerning are life-threatening arrhythmias such as torsades de pointes [9]. Neurologic dysfunction secondary to hypomagnesemia can manifest in the form of tremors, weakness, tetany, and convulsions [10]. Most patients become symptomatic below a serum magnesium level of 1.2 mg/dL, but this is not always the case, as in our patient [11]. This highlights the need for serial monitoring of magnesium levels in patients on PPI, especially those with additional ongoing renal or gastrointestinal losses. A study on mice concluded that both treatment with PPI (omeprazole) and low dietary magnesium intake can cause alterations in the lower gut microbiota, resulting in low serum magnesium [12]. This effect is independent of any secondary effect on gastrointestinal or renal magnesium transporters. Another remarkable feature of this case was the absence of other common causes of hypomagnesemia, such as chronic alcohol use or diuretic therapy. This highlights the importance of patient education surrounding measuring stoma output, dietary advice, and seeking medical attention for a high-output stoma.

This patient presented with recurrent, profound hypomagnesemia, which can lead to life-threatening complications. This is despite daily oral magnesium supplementation. A low FeMg of <2% was in keeping with non-renal losses of magnesium. The most likely route for this was the GI tract. The patient was counseled on lifestyle changes for the management of his GERD, and an H2-receptor antagonist was prescribed for use on an as-needed basis. This resulted in the resolution of his recurrent hypomagnesemia and the elimination of the need for magnesium supplementation. PPI-induced hypomagnesemia is reversible with resolution of hypomagnesemia on withdrawal of PPI therapy; however, depletions recur on reintroduction of PPI therapy [6].

## Conclusions

The use of proton pump inhibitors can result in severe hypomagnesemia in specific settings, which can have significant cardiovascular and neurologic implications. This case highlighted the consequences of PPI combined with ongoing gastrointestinal losses with an ostomy. Patients who are on daily PPI therapy may not have routine laboratory investigations for several months and are at risk for these electrolyte derangements, which increase their risk of fatal cardiac arrhythmias. Routine use of proton pump inhibitors in patients susceptible to hypomagnesemia should be regularly reviewed with laboratory monitoring and used only when another alternative treatment is not available.
